# Payment for performance (P4P): any future in Italy?

**DOI:** 10.1186/1471-2458-11-377

**Published:** 2011-05-24

**Authors:** Silvana Castaldi, Annalisa Bodina, Luciana Bevilacqua, Elena Parravicini, Michaela Bertuzzi, Francesco Auxilia

**Affiliations:** 1 Department of Public Health, Microbiology and Virology, University of Milan, Italy; 2 Fondazione IRCCS Ca' Granda Ospedale Maggiore Policlinico, Milan, Italy; 3 AO Ospedale Niguarda Ca' Granda, Milan, Italy

**Keywords:** performance, quality, payment, measurement

## Abstract

**Background:**

Pay for Performance (P4P) programs, based on provision of financial incentives for service quality, have been widely adopted to enhance quality of care and to promote a more efficient use of health care resources whilst improving patient outcomes. In Italy, as in other countries, the growing concern over the quality of health services provided and the scarcity of resources would make P4P programs a useful means of improving their performance. The aim of this paper is to evaluate whether it is possible to implement P4P programs in the Lombardy Region, in Italy, based on the existing data set.

**Methods:**

Thirteen quality measures were identified regarding four clinical conditions (acute myocardial infarction (AMI), heart failure (HF), ischemic stroke and hip and knee replacement) on the basis of an international literature review. Data was collected using the database of three institutions, which included hospital discharge records (Scheda di Dimissione ospedaliera-SDO-) and letters of discharge. The study population was identified using both the Principal ICD-9-CM diagnosis codes and the discharge date. A Statistical Analysis System (SAS) program was used for the text analysis.

**Results:**

It was possible to calculate almost all the parameters pertaining to the three hospitals as all the data required was available with the exception of inpatient mortality in two hospitals and smoking cessation advice/counseling in one hospital.

**Conclusions:**

On the ground of this analysis, we believe that it is possible to implement a P4P program in the Lombardy Region. However, for this program to be initiated, all necessary data must be available in electronic format and uniformly collected. Moreover, several other factors must be assessed: which clinical conditions should be included, the threshold for each quality parameter, the amount of financial incentives offered and how they will be provided.

## Background

Escalating costs and the growing imbalance between primary and specialty care, have highlighted the urgent need for a deep reform of the health care payment system. One of the core problems of the existing system is that the dominant fee-for-service model rewards volume and intensity rather than value and quality of care. Although the faults of the existing healthcare payment system are evident, there is great uncertainty as to which approach would achieve the best results[1].

In the United States of America (USA), performance based remuneration programs ("Pay for Performance"-P4P) for general practitioners and hospitals have been experimentally introduced since 2001, in an effort to better reward service quality[2].

Earlier this decade, pay for performance (P4P) programs took center stage as a tactic for realigning payment with value. P4P programs aim to overcome the limits of the fee-for-service model based on DRG (Diagnosis Related Group) fixed rate remuneration, by paying extra for the implementation of specific processes/procedures necessary to enhance patient outcome. Two types of information are required for the correct operation of a P4P program. These include health process and outcome measures that can be positively influenced by clinical management and information about specific treatments and clinical services that can improve an individuals' poor health status (health production function)[3].

The P4P initiative in healthcare management is based on the concept of fostering and rewarding improvement in this sector. Most P4P programs are designed to promote value-based health care, defined as a more effective distribution of funds and efforts through measurement, transparency and accountability[4]. The basic idea behind this initiative is to provide healthcare providers with financial incentives to achieve specific quality standards[5]. In other words, this payment model rewards physicians, hospitals, medical groups and other healthcare providers for meeting certain quality and efficiency performance measures, which the providers and purchasers have previously agreed in writing.

The popularity of P4P programs can be attributed to the desire to explore new payment methods that can improve quality and reduce costs, based on the hypothesis that, in healthcare, as in other industries, higher quality equals lower cost[6]. In healthcare, the relationship between high quality and low cost has yet to be fully demonstrated. The ultimate goal of P4P reforms is to provide evidence-based care and to ensure that process management is supplemented by patient-reported outcome measures[7]. In the early 1970s, the British epidemiologist Archie Cochrane considered that, because of limited societal resources, only healthcare services shown to be effective should be provided to patients[8].

P4P is increasingly used to improve healthcare quality and safety[9]. More than half of all commercial health maintenance organizations in the USA employ a P4P program, and approximately 40 P4P programs focus specifically on inpatient hospital care[10,11]. P4P has been implemented in other countries (for instance, P4P incentives were introduced in the UK for primary care in 1991[12]) and more countries are considering their introduction. As was the case with the DRG and quality certification systems, there is reason to believe that P4P programs will eventually be introduced in Italy as well.

The purpose of this paper is to evaluate whether data and conditions for the implementation of P4P programs exist in Lombardy, in the north of Italy. We focused our attention on inpatient hospital care.

## Methods

Based on an international literature review (done through a search in PubMed of the terms: P4P, quality program, outcome, payment), thirteen quality parameters were identified regarding four clinical conditions: acute myocardial infarction (AMI), heart failure (HF), ischemic stroke and hip and knee replacement (table 1)[1,3,9,10,13-23]. Data on these thirteen parameters were available in electronic format.

**Table 1:** Quality measures (n = 13) and clinical conditions (n = 4) used in the present study.

Clinical Conditions	Measures
Acute Myocardial Infarction (AMI)	1. Aspirin prescribed at discharge

	2. Beta-blocker prescribed at discharge

	3.Adult smoking cessation advice/counseling

	4. LDL cholesterol assessment

	5.Lipid lowering therapy at discharge

	6. Inpatient mortality

Heart Failure (HF)	7.Evaluation of left ventricular systolic (LVS) function (LVF Assessment)

	8. ACEI or ARB for left ventricular systolic dysfunction (LVSD)

	9. Adult smoking cessation advice/counseling

Ischemic Stroke	10. Thrombolytic therapy administered

	11. Discharged on cholesterol reducing medication

	12. Smoking cessation advice/counseling

Hip and Knee Replacement	13.Recommended Venous Thromboembolism (VTE) Prophylaxis Ordered

Data were collected from three public hospitals of national interest. We chose these three hospitals from over one hundred other options in the Lombardy Region because they were the first to comply with all the IT standards set by the Regional Health Authority. Therefore, these three hospitals are not representative of the health structure in the entire Region of Lombardy, but were selected because they already reached the IT standard that all hospitals will be expected to achieve in the near future. Data sources include two formal statutory documents available in electronic format: hospital discharge records (Scheda di Dimissione ospedaliera-SDO-) and letters of discharge. It is important to assess whether the quality controls carried out by the Region of Lombardy show that the coding method employed in the three hospitals is accurate (i.e. giving patients a diagnosis) and that physicians attend mandatory training courses for completing letters of discharge.

No ethical approval is required for this study according to the Italian law 196/2003 and according to Regolamento Regionale Lombardo n.9, july 2006 (BURL n.29, II suppl. Ord. 21/07/2006); the study only involved the use of anonymized data already available at the hospitals.

The study population was identified using two data elements: the Principal ICD-9-CM diagnosis codes and the discharge date. All patients discharged in 2008 were considered eligible. Patients who died while in hospital were excluded from all measurements, with the exception of inpatient mortality. In addition, nonsmokers and former smokers were excluded from the "smoking cessation counseling" measure.

Measures are expressed as the number of times a selected procedure (i.e. Beta-blockers at discharge) was performed in eligible patients at a selected hospital, divided by the total number of patients eligible for that procedure and treated at that hospital. In order to ascertain whether a selected procedure has been performed in eligible patients, a list of key words was devised for each measure and a search was performed on the letters of discharge. For instance, in order to find out the percentage of patients with AMI who were prescribed beta-blockers at discharge, a list of beta-blockers agents was created, including both active pharmaceutical ingredients and trade names, together with employed abbreviations and acronyms. These words were searched for in all letters of discharge.

The Statistical Analysis System (SAS) program, and, in particular, Base SAS Software, were used for the text analysis of discharge letters. In particular, the "index" function, which searches for a text string inside a database, has been employed. The "upcase" function was used to include both upper and lower case text.

The results obtained from the automatic search performed on the letters of discharge were then checked manually and ratings of "true/false positive" and of "true/false negative" assigned. Finally, the sensitivity and specificity of the method were calculated. [Sensitivity = number of true positives/(sum of true positives + false negatives); Specificity = number of true negatives/(sum of true negatives + false positives)].

## Results

The number of patients included in the different analyses of hospital performance on process measures is shown in Table 2.

**Table 2:** Number of patients included in the analyses of hospital performance on process measures.

	AMI		HF		Ischemic Stroke		Hip/Knee Replacement	
	
	Patients	Letters of discharge	Patients	Letters of discharge	Patients	Letters of Discharge	Patients	Letters of Discharge
Hospital N.1	498	460	1590	1590	347	347	182	182

Hospital N.2	557	345	1761	1277	572	420	533	533

Hospital N.3	325	305	575	575	172	172	200	200

The results for each measure are summarized in Tables 3 and 4. There is missing data for hospitals n. 2 and n. 3 in the inpatient mortality measure and for hospital n. 2 in the adult smoking cessation advice/counseling measure. Mortality rate data was not available, while it was not possible to calculate the rate in adult smoking cessation advice/counseling data because the structure of the letter of discharge electronic format did not allow us to identify smokers among eligible patients or smoking cessation advice in discharge recommendations.

**Table 3:** Number of eligible patients and number of times a selected procedure was performed in eligible patients.

	Hospital N.1		Hospital N.2		Hospital N.3	
	
	N. of times the selected procedure is performed in eligible patients	N. of eligible patients	N. of times the selected procedure is performed in eligible patients	N. of eligible patients	N. of times the selected procedure is performed in eligible patients	N. of eligible patients
1. Aspirin prescribed at discharge	390	460	323	345	262	305

2. Beta-blocker prescribed at discharge	376	460	249	345	200	305

3. Adult smoking cessation advice/counseling	16	139			2	46

4. LDL cholesterol assessment	73	460	48	345	1	305

5. Lipid lowering therapy at discharge	376	460	256	345	219	305

6. Inpatient mortality	38	498				

7. Evaluation of left ventricular systolic (LVS) function (LVF Assessment)	1049	1590	663	1277	314	575

8. ACEI or ARB for left ventricular systolic dysfunction (LVSD)	1059	1590	909	1277	352	575

9. Adult smoking cessation advice/counseling	22	145			6	33

10. Thrombolytic therapy administered	15	347	6	420	0	172

11. Discharged on cholesterol reducing medication	74	347	174	420	37	172

12. Smoking cessation advice/counseling	4	33			8	10

13. Recommended Venous Thromboembolism (VTE) Prophylaxis Ordered	169	182	0	533	24	200

**Table 4:** Results for each measure.

Measures	Hospital N.1	Hospital N.2	Hospital N.3
1. Aspirin prescribed at discharge	84.8%	93.6%	85.9%

2. Beta-blocker prescribed at discharge	81.7%	72.2%	65.6%

3 Adult smoking cessation advice counseling.	11.5%	4.3%	4.3%

4. LDL cholesterol assessment	15.9%	13.9%	0.3%

5. Lipid lowering therapy at discharge	81.7%	74.2%	71.8%

6. Inpatient mortality	7.6%		

7. Evaluation of left ventricular systolic (LVS) function (LVF Assessment)	66.0%	51.9%	54.6%

8. ACEI or ARB for left ventricular systolic dysfunction (LVSD)	66.6%	71.2%	61.2%

9. Adult smoking cessation advice counseling	15.2%	18.2%	18.2%

10. Thrombolytic therapy administered	4.3%	1.4%	0.0%

11. Discharged on cholesterol reducing medication	21.3%	41.4%	21.5%

12. Smoking cessation advice/counseling	12.1%		80.0%

13. Hip or Knee replacement Patients with Recommended Venous Thromboembolism (VTE) Prophylaxis Ordered	92.9%	0.0%	12.0%

The sensitivity and specificity of the method, for each measure analyzed, are summarized in Table 5. Specificity ratings reached 100% in the majority of cases, meaning that the test effectively excluded all negative readings (i.e. if beta-blockers are mentioned, they were certainly referred to in the letter). As for sensitivity, though the ratings did not always reach 100%, scores were considered acceptable as sensitivity values always exceeded 90%, with the exception of former smokers in patients with HF and LDL cholesterol admitted at hospital n.3.

**Table 5:** Sensitivity and specificity of the method used.

	Hospital N.1		Hospital N.2		Hospital N.3	
	
	Sensibility	Specificity	Sensibility	Specificity	Sensibility	Specificity
1. Aspirin prescribed at discharge	96.1%	100.0%	99.7%	100.0%	97.8%	100.0%

2. Beta-blocker prescribed at discharge	95.7%	100.0%	97.6%	100.0%	100.0%	100.0%

3. Adult smoking cessation advice Counseling	100.0% (68.9% for the research of Former smokers)	100.0%			100.0% (100.0% for the research of former smokers)	100.0%

4. LDL cholesterol assessment	100.0%	100.0%	100.0%	100.0%	33.3%	100.0%

5. Lipid lowering therapy at discharge	100.0%	100.0%	100.0%	100.0%	100.0%	100.0%

6. Inpatient mortality	100.0%	100.0%				

7. Evaluation of left ventricular systolic (LVS) function (LVF Assessment)	99.0%	100.0%	100.0%	100.0%	100.0%	100.0%

8. ACEI or ARB for left ventricular systolic dysfunction (LVSD)	100.0%	100.0%	100.0%	100.0%	100.0%	100.0%

9. Adult smoking cessation advice counseling	96.3% (94.5% for the research of former smokers)	100.0%			100.0% (73.3% for the research of former smokers)	100.0%

10. Thrombolytic therapy administered	100.0%	100.0%	100.0%	99.5%	100.0%	100.0%

11. Discharged on cholesterol reducing medication	100.0%	100.0%	100.0%	100.0%	100.0%	100.0%

12. Smoking cessation advice/counseling	100.0% (100.0% for the research of former smokers)	100.0%			100.0%	97.6% (100.0% for the research of former smokers)

13. Hip or Knee replacement Patients with Recommended Venous Thromboembolism (VTE) Prophylaxis Ordered	99.4%	100.0%	100.0%	100.0%	100.0%	100.0%

## Discussion and Conclusions

The aim of this paper is to evaluate whether data and conditions for the implementation of P4P programs exist in the Region of Lombardy, Italy. Three hospitals of national interest were chosen as sites where 13 outcome measures relating to four clinical conditions have been evaluated. This set of indicators was chosen since it was considered to be reasonably applicable to hospitals in the Region of Lombardy.

In order to better assess the results obtained, several specific considerations should be made for a number of indicators used in the evaluation:

- Aspirin prescribed at discharge: results had shown that the drug was prescribed at discharge in more than 80% of cases of AMI. However, it is possible that discharge records that do not include the prescription of this drug, relate to patients who were already on anticoagulation therapy.

- Beta-blocker prescribed at discharge: beta-blockers were prescribed at discharge in more than 65% of cases of AMI in all three hospitals. The data must be assessed taking into account the relative contraindications associated with the use of beta-blockers. There are many contraindications including asthma and reversible airway obstruction in patients with respiratory diseases, atrioventricular conduction disturbances, severe bradycardia, Raynaud's phenomenon and a clinical history of depression[24]. A manual check of the files revealed that in many cases in which beta-blockers were not prescribed, patients were already receiving beta-agonists treatment for respiratory diseases.

- Lipid lowering therapy at discharge: the list of drugs used for the calculation of the indicator included all the active pharmaceutical ingredients used to lower blood lipids (triglycerides and/or cholesterol) and improve lipid profile (increased HDL cholesterol). However, a more stringent selection of active ingredients would be necessary for a more specific goal (eg reduction of LDL cholesterol). In any case, the indicator should be assessed taking into account that no distinction has been made between AMI types. A better level of accuracy would be guaranteed by identifying AMI patients who are hyperlipidemic and limiting the lipid lowering therapy assessment to this specific sub-group.

- Thrombolytic therapy: the available data did not allow the selection of ischemic stroke patients who were eligible for thrombolytic therapy. Therefore, all patients diagnosed with ischemic stroke were considered eligible. In the perspective of introducing a P4P program this assumption should be modified.

In summary, since this was a first attempt of calculation, a simple, although less accurate measuring system was applied. Adjustments would be required if a P4P system was implemented in the Region of Lombardy.

The results obtained for each measure can be considered a starting point for the target definition. In order to implement a P4P program, it is necessary to determine which level of performance will merit a financial incentive. However, at present, this decision is made difficult by the considerable variability in the performance of the three hospitals.

It would be interesting to compare the performance of these three hospitals with the averages obtained for other health systems, but this is not the aim of the study. Furthermore, in order to make this comparison we would need to define the selection criteria of the patient group studied and all hospitals must have electronic data available.

Electronic data sources were used (i.e. letters of discharge). Hard copy data sources were not considered in the present evaluation due to the additional cost burden on the P4P program. In fact, information on P4P program measures needs to be easily accessible. All three chosen hospitals had letters of discharge in electronic format. In the course of the evaluation, it became apparent that not all the information required for the selected measures was available in the letters of discharge. Data on venous thromboembolism prophylaxis was not reported by one of the three hospitals analyzed. In addition, there is indication that several letters were incomplete; for example, only 46 patients with AMI and 10 patients with ischemic stroke admitted to hospital n. 3 were reported as smokers. This information gap may be due to the fact that until now these details were never requested or subject to evaluation. As has been the case in the USA, the introduction of a P4P program is likely to result in rapid improvement in the reporting of these measures, primarily thanks to improved documentation of clinical activities [10,21-23,25-27]. Clinical data will continue to be difficult to obtain, especially in the absence of widespread use of electronic record supports, but the measurement may offer the opportunity to train physicians to comply with guidelines and to document their work better.

During the past decade, the use of hospital-based P4P programs to improve quality, has largely expanded. However, few programs were systematically evaluated, leaving several substantial gaps in the knowledge of their effectiveness [9,28]. First, the clinical conditions used as a basis for P4P systems have so far been limited to cardiovascular disease, pneumonia, hip and knee replacement and few others. Therefore, there is virtually no knowledge about the effects of P4P incentives applied to other conditions. Second, the effect of different types of incentives used in hospital P4P programs is unknown. Third, there is little evidence to assess the effect of P4P programs on quality. Fourth, it is yet unknown whether the quality improvements resulting from hospital P4P programs outweigh their cost.

This paper demonstrates that a P4P program could be feasible in the Region of Lombardy. However, solid electronic information systems should be put in place in order to contain implementation costs and to enable the rapid and effective calculation of measures. All physicians in hospitals must be trained to select patients data correctly, which they have to enter in the patient's electronic records. The data obtained from the three hospitals we studied, where this process has started, shows good results, the data entered and its quality may allow Regional Health Authorities to implement this program.

In conclusion, all data necessary for P4P programs should be available in electronic format and uniformly collected before these programs are introduced. Several other factors that should be clarified prior to implementing P4P programs in the Region of Lombardy and subsequently in Italy, include: which clinical conditions should be included, the threshold for each quality measure, the amount of financial incentives offered and how they will be provided.

No ethical approval was necessary because the study only involved anonymized data available on request from each hospital.

## Competing interests

The authors declare that they have no competing interests.

## Authors' contributions

SC participated in the design of the study and in its coordination. AB participated in the design of the study, performed the analysis and drafted the manuscript. LB participated in the design of the study. EP participated in the design of the study and performed the analysis. MB performed the analysis. FA participated in the design of the study. All authors read and approved the final manuscript.

## Pre-publication history

The pre-publication history for this paper can be accessed here:

http://www.biomedcentral.com/1471-2458/11/377/prepub
